# Supervised Learning with Complex Spikes and Spike-Timing-Dependent Plasticity

**DOI:** 10.1371/journal.pone.0099635

**Published:** 2014-06-19

**Authors:** Conor Houghton

**Affiliations:** Department of Computer Science, University of Bristol, Bristol, England; University of Sheffield, United Kingdom

## Abstract

One distinctive feature of Purkinje cells is that they have two types of discharge: in addition to simple spikes they fire complex spikes in response to input from the climbing fibers. These complex spikes have an initial rapid burst of spikes and spikelets followed by a sustained depolarization; in some models of cerebellar function this climbing fiber input supervises learning in Purkinje cells. On the other hand, synaptic plasticity is often thought to rely on the timing of pre-synaptic and post-synaptic spikes. It is suggested here that the period of depolarization following a complex spike, combined with a simple spike-timing-dependent plasticity rule, gives a mechanism for the climbing fiber to supervise learning in the Purkinje cell. This proposal is illustrated using a simple simulation in which it is seen that the climbing fiber succeeds in supervising the learning.

## Introduction

In addition to weak inputs from parallel fibers and local inhibitory cells, a Purkinje cell receives a strong input from a single climbing fibre [1], [2]. The Purkinje cell has two distinct types of spike; simple spikes, in response to the aggregated signal from many weak inputs from the parallel fibers, and complex spikes, in response to an input from the climbing fiber. The simple spikes are similar to the spikes found throughout the nervous system whereas complex spikes have a distinctive structure with a leading spike followed by a series of partial spikes called spikelets and a sustained period of depolarization during which the cell is refractory [2]–[6].

It has been known for nearly two hundred year that the cerebellum is important for motor coordination [7], [8] and, while there are non-motor symptoms [9], [10], damage to the cerebellum is most closely associated with motor problems: intentional tremor, a want of harmony in movement, a loss of kinetic melody, a distinctive wide stance and an unsteady ataxic gait [11]–[14]. However, the precise role of the cerebellum in movement is still debated; it is proposed, for example, that the cerebellum is a computational engine for deciding precise levels of muscle activation [17]–[20], or an organ of prediction, predicting the sensory consequence of movement [21]–[24] or that its has a role in proprioception [25], [26]. Of course, these putative functions may co-exist, or may be co-dependent, but there is no definitive description of what the function of the cerebellum is and there certainly no theory of cerebellar function which would predict its distinctive structure.

There are also many compelling ideas as to what the role of complex spikes may be. Some ideas relate to signalling: it has been proposed, for example, that complex spikes may provide a timing signal for motor control, [15], [16]. Other ideas relate to learning; in the Marr-Albus-Ito model of the cerebellar cortex, climbing fiber spikes are a supervisory error signal [17]–[19], [27]. Similarly, in models where the cerebellum is seen as predicting sensory outcomes, the climbing fiber input communicates significant deviations from the sensory prediction, a deviation which is incorporated into future predictions, if it is repeated with sufficient frequency. As with the overall function of the cerebellum, the complex spike is likely to subserve multiple functions. However, there is evidence that acting as learning signal is one these function: an increase in the frequency of complex spiking is associated with depression in the synapses connecting the parallel fibers to the Purkinje cells and a decrease is associated with potentiation of those synapses [28]–[30]. This paper proposes a mechanism for this plasticity. Long term plastic changes to these synapses are likely to be mediated by rather complex mechanisms. Here, however, a very simple mechanism is proposed, it is suggested that the refractory pause caused by a complex spike plays a crucial role in plasticity.

This proposal is made in the context of spike-timing-dependent plasticity (STDP). STDP describes models of long term plasticity in which the changes to synaptic strengths depend on millisecond-scale differences in spike times. In STDP models changes to synapse strengths depend on the timing difference between pre-synaptic and post-synaptic spikes. Frequently for excitatory synapses, this dependence is anti-symmetric, so, if a pre-synaptic spike precedes a post-synaptic one the synapse is potentiated, but if the order is reversed, the synapse is depressed. In other words, long term depression (LTD) and potentiation (LTP) depend on the timing of pre and post-synaptic spikes as well as the coincident activity of the pre and post-synaptic neurons.

A causal structure was implicit in the original formulation of Hebbian plasticity [31], a remarkable piece of prescience: in the nineties a series of papers pointed to experimental evidence for timing effects in plasticity [32]–[36] including a definitive demonstration of a spike-order dependent plastic changes *in vitro* in [33], the observation of asymmetric STDP *in vivo* in developing Xenopus in [37] and a clear graph of the time dependence of plastic changes *in vitro* in [38]. STDP has also been observed *in vivo* in mammal in, for example, [39]–[43] and, in fact, STDP has now been observed across animal models, brain regions and cell types. The asymmetric causal window is the most common STDP rule, but there is considerable variability, sometimes even within the same neuron, for a review see [45]; [44] gives an historical account.

Interest in putative functional roles for STDP preceded biological evidence for it [46] and a wide range of possible roles are reviewed in [47]. One particular strength of STDP rules is that they are capable of doing unsupervized learning of spike-spike correlations in the input [48]–[50]. Roughly speaking, if spikes from a group of pre-synaptic neurons tend to arrive at roughly the same time, they are more likely to cause a post-synaptic spike, resulting in potentiation.

**Figure 1:**
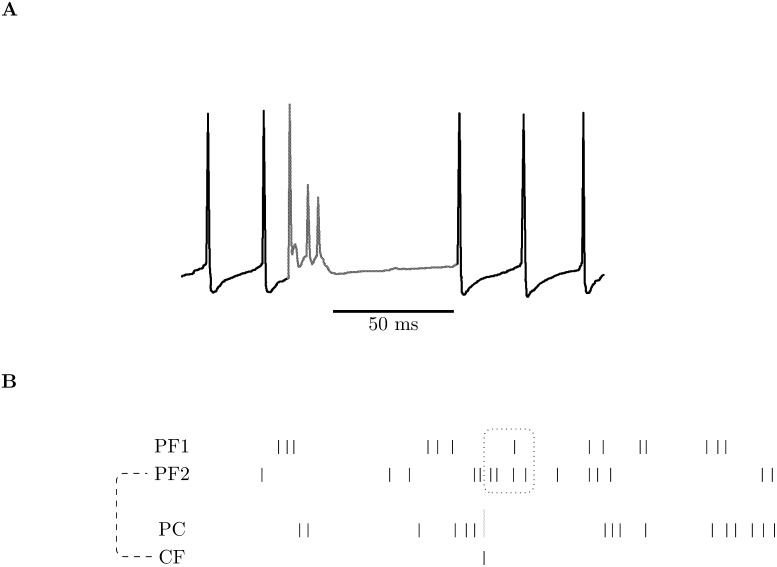
Complex spikes and STDP. **A** gives a sketch of a complex spike with its characteristic spikelets and extended refractory period. **B** gives a cartoon introduction to the supervized learning scheme introduced in this paper. PC marks the Purkinje cell spike train, PF1 and PF2 mark two parallel fiber spike trains and CF the climbing fiber spike train. As indicated by the dashed line, PF2 and CF are correlated and the only climbing fiber spike in this period occurs during a burst of spikes from PF2. This causes a complex spike in PC, marked by a tall green line. The spikes in the dotted box are anti-causal to the complex spike and close to it in time. As a consequence, will cause LTD in the corresponding synapses with a greater amount of depression in the PF2 synapse, since it has more spikes in the dotted box.

It is proposed here that the learning role of complex spikes is to give a pause during which there is no post-synaptic spike. This will mean any pre-synaptic spikes will be in an anti-causal relationship with the initial spike of the complex spike. According to the standard STDP rule, this will cause long-term depression. In short, it is proposed that a standard STDP rule governs plasticity in synapses from the parallel fibres and local inhibitory neurons and the combination of this rule and the refractory pause caused by the complex spike results in supervize learning. This is broadly in line with the models of cerebellar function in which climbing fiber supervises learning by depressing synapses: this model incorporates supervized learning in response to an error signal carried by the climbing fibre. In fact, the proposal described here can be thought of as a putative mechanism for implementing this aspect of cerebellar models. Figure 1 gives a schematic of this proposal and a simple simulation is introduced below to examine the proposal.

## Methods

**Figure 2:**
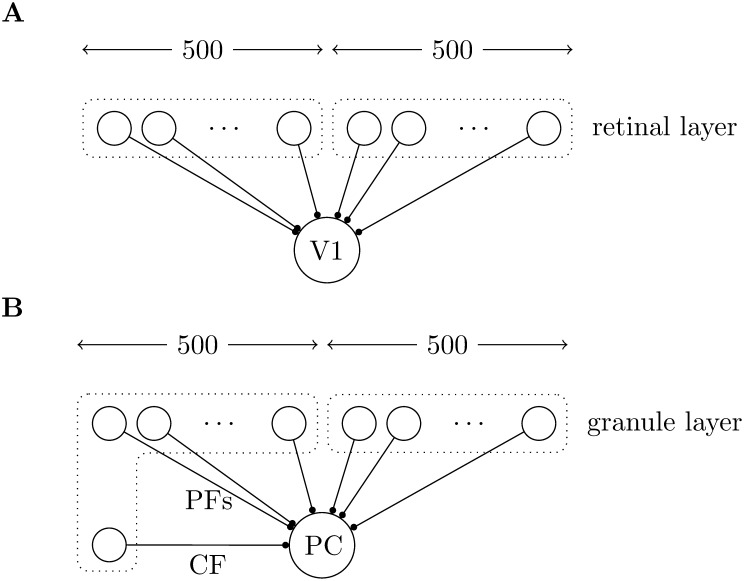
A summary of the model. **A** shows the network used in [50]; 1000 retinal layer neurons feed forward to a V1 neuron. The V1 neuron is modelled as an integrate and fire neuron without refractory period. The retinal layer neurons are modelled as Poisson processes; they are divided into two equal groups and the firing rates in each group are correlated with each other, but not with the firing rates for neurons in the other group. **B** shows the same model adapted to the cerebellum, what was the retinal layer is now the granule layer so their feed forward projections are now parallel fibers (PFs), the V1 neuron is now a Purkinje cell (PC). There is an extra neuron whose projection models the climbing fiber (CF); its firing rate is correlated to the firing rates in one of the two groups of granule cells but the effect of spikes in the climbing fiber are different, it causes the Purkinje cell to spike and then undergo a refractory period.

The simple model used here is based directly on the one used in [50] to demonstrate the learning of spike-spike correlation structure in STDP. In that paper, two network models of the sensory system are considered. In each of these there is an input layer, which will be referred to as *the retinal layer*, feeding forward to a processing layer. In one model the processing layer has only one neuron, in the other the processing layer is itself a recurrent network. The simpler model, with a single processing layer neuron, is considered here. The single neuron is referred to as the *V1 neuron*. This model is illustrated in Fig. 2**A**.

There are 1000 retinal layer neurons which are modelled using Poisson spiking. As in the figure, Fig. 2, these all feed forward to the single V1 neuron. Each neuron in the retinal layer has a firing rate; this firing rate is step-wise constant: each neuron has a fixed constant value for an interval, but this value changes from interval to interval. The firing rates are chosen so that there are two groups of retinal layer neurons, indicated by the dotted boxes in the figure. The firing rates inside each group are correlated with each other, but not with neurons in the other group. This is described in more detail below.

The V1 neuron is modelled as an integrate and fire neuron with no refractory period. Its voltage 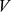 satisfies
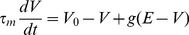
(1)where 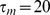 ms is the membrane time constant, 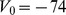 mV is the resting potential and 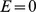 mV is the reversal potential for the synaptic current. If 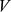 reaches the threshold value 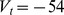 mV the neuron spikes and 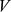 is reset to 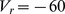 mV. 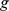 is the total synaptic conductance calculated by summing over the individual conductances of the synapses from the retinal neurons
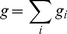
(2)where each synapse connecting a retinal neuron to the V1 neuron has a conductance 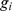 which satisfies
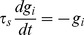
(3)with 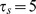 ms. Whenever there is a pre-synaptic spike the conductance increases instantaneously by an amount 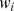:
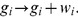
(4)This 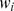 can be thought of as the synapse strength which may be changed by synaptic plasticity.

To implement the STDP rule each synapse has two potentials [47]. One potential, 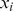, increases in response to pre-synaptic spikes and satisfies
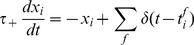
(5)where 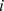 labels the spike time and the pre-synaptic spike times are labelled 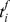. The other potential, 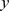, increases in response to post-synaptic spikes and satisfies
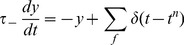
(6)where the postsynaptic spike times are 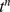. The evolution of the synapse strength is now
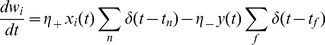
(7)with hard lower and upper bounds on 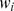:
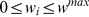
(8)where 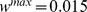.

The choice of 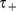 and 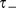 effects the temporal extent of the correlation that can be learned; here 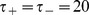 ms. The choice of 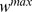 sets a synaptic scale. 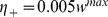 and 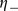 is set so that
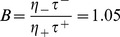
(9)which means 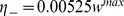 and is larger than 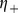 so if there is no relationship between pre- and post-synaptic spikes at a synapse its strength falls.

Thus, the STDP used here is additive with an exponentially decaying plasticity window and, in a biologically appropriate way, the individual spike-spike pairs are not tabulated, rather, a running account is maintained using potentials. The ability of this version of the STDP to learn structure in the input data is demonstrated in [50] by dividing the retinal neurons into two correlated groups.

The retinal neurons are divided into two equal sized groups with the firing rates for each neuron chosen randomly, but in such a way as to give correlated firing inside each group. Specifically, for each group all the neurons are given a firing rate
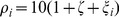
(10)where both 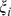 and 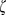 are chosen from normal distributions with mean zero and variance one. While the same 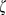 is used for all neurons in the group, each 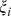 is chosen separately. These firing rates are held fixed for a time interval chosen randomly using an exponential distribution with rate 50 Hz, this corresponds to an average interval length of 20 ms, with the intervals determined separately for each group. At the start of each new interval a new 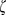 and new 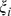 are chosen, giving the rates, 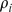, for the neurons in the group.

One of the two groups of neurons wins out over the other group. Small initial variations give one group a slightly greater tendency to control the post-synaptic neuron. The synapses in this group get stronger, reducing the proportion of post-synaptic spikes that are in a causal relationship with the pre-synaptic spikes from the other group. This means that the strength of these synapses will diminish. This process is slow, but with time, one group has synapses with strengths close to zero, the other, strengths close to 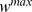.

This Song-Abbott model exhibits unsupervised learning; the STDP rule allows the simple network to learn a correlation pattern in the input, but it does not determine which pattern it learns. Small initial differences decide which of the two groups wins out. The idea here is to show how a neuron modelled on the climbing fiber in the cerebellar cortex can act as a supervisor and determine which group ends up with stronger synapses.

The goal here is to illustrate the proposed learning mechanism using the network from [50] described above. To illustrate the proposal a single extra neuron has been added to the existing simulation. To make it easy to compare to the original model, nothing else has been changed, however, the pre-synaptic layer neurons are now interpreted as corresponding to parallel fibers and the post-synaptic neuron is interpreted as being a Purkinje cell. This is shown in Fig. 2**B**: the input later neurons are now considered to model granule cells rather than retinal cells, the V1 neuron has been replaced by a Purkinje cell and there is now a climbing fiber whose firing rate puts it in one of the two groups of granule cells.

**Figure 3:**
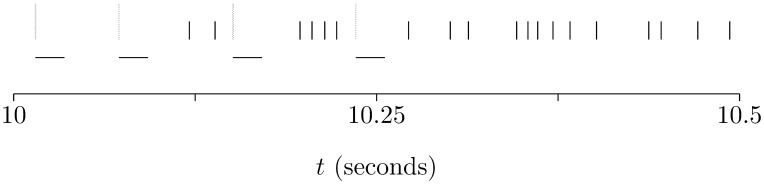
An example of spiking activity in the model. This shows the spiking activity in the Purkinje cell for one simulation trial; the activity between 10 s and 10.5 s is given, in other words, it shows the activity before plasticity has had any substantial effect on the behavior of the cell. The simple spikes are marked with short black vertical dashes, the complex spikes by tall green vertical dashes; the horizontal dashes mark the refractory periods which follow each complex spike. During these period 481 spikes arrive from the input neurons correlated with the climbing fiber activity and 354 spikes from the other group.

Thus there is now an extra input, the climbing fiber is considered to belong to one of the two groups of neurons in the pre-synaptic layer; its rate is set at 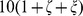 where 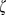 is the same value chosen for the other neurons in its group and 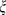 is chosen specifically for this neuron from the usual normal distribution. In this way, this neuron resembles the other pre-synaptic neurons in its own spiking, however, its effect on the post-synaptic neuron is very different: if the climbing fiber neuron fires so does the post-synaptic neuron and, after it fires, the conductance in the post-synaptic neuron is fixed at zero for the refractory period of 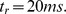 This mimics a complex spike. Figure 3 shows an example of spiking activity in the model.

## Results

**Figure 4:**
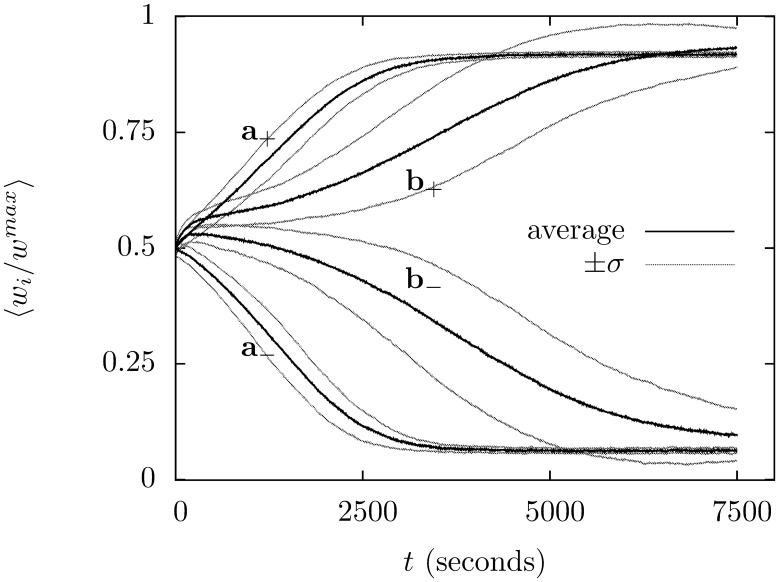
The progress of supervised learning in the supervised and unsupervised cases. The value of 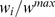 is averaged across each group during the simulation; the simulation is repeated for 100 trials and averaged. The curves for the supervised learning are marked 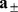 with 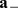 indicating the group correlated to the climbing fiber. In the case of the unsupervised learning the group that wins out varies from trial to trial, but the average is taken across matching groups: 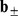 mark the unsupervized learning groups, with 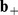 the average for the group that ends up with the larger average value. The black lines trace the average, the grey lines show the size of trial-to-trial variation, marking 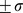.

The climbing fiber succeeds in supervising the learning, in every one of a 100 trials the group with the climbing fiber is the one that ends up with very low synapse strengths. This shows that the supervision completely lifts the ambiguity in which group ends up with low synaptic strengths and which ends up with high. Supervised learning is also much faster. In Fig. 4 the average value of 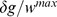 for the two groups is plotted against time. The supervised learning reaches an equilibrium after 2500 seconds but the unsupervised learning only reaches it after 7500 seconds.

Averaged over 100 trials the group correlated with the climbing fibre has 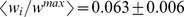 after 7500 s; the other group has final value 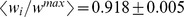 where the plus-or-minus gives the standard deviation. The same values for unsupervized learning are 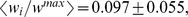 averaged over the group that ends up lower and 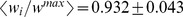 for the other group. Thus, the averages are similar but the variation greater; however, the most significant difference is that supervision increases the speed of learning and determines which group is which.

**Figure 5:**
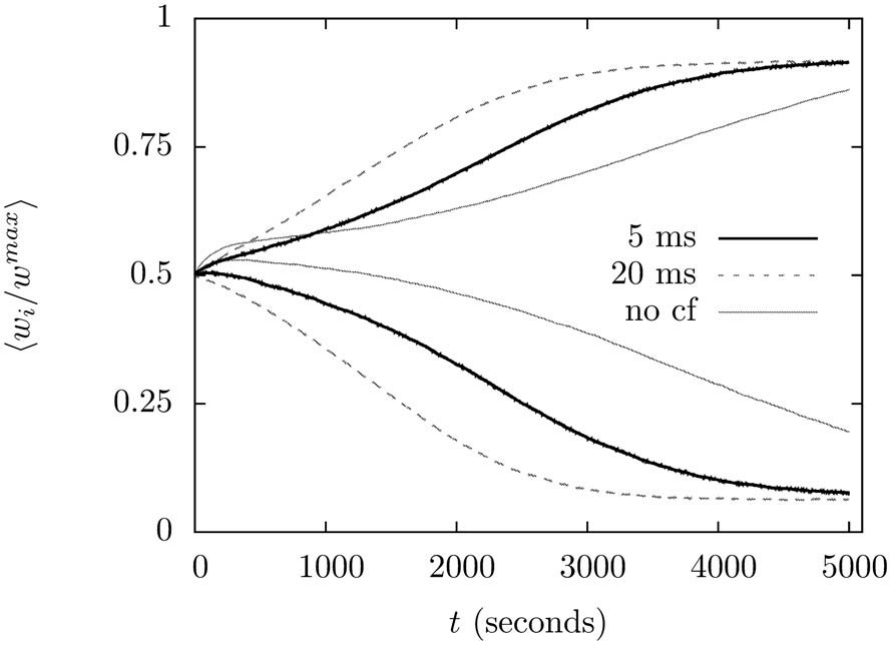
The effect of shortening the refractory period following a complex spike. The solid black line graphs the synapse strengths for the two groups in a simulation where the refractory period following climbing fiber input is reduced to 5 ms. As before, the result has been average over 100 trials. Learning is still effective in the sense that the same group always wins; learning is, however, slower. For comparison, the same graph for the original refractory period of 20 ms has been include in dashed grey and the graph for unsupervized learning in solid grey.

The success of supervision is robust, it does not rely on a precise choice of climbing fiber parameters. The rate of learning does become slower if the refractory component of the complex spike is reduced, for example. Fig. 5 shows the effect of reducing the refractory period to 5 ms, the learning remains reliable but is slower to reach equilibrium. Increasing the refractory period to 40 ms has no discernible effect on learning.

To investigate robustness of the supervised learning another simulation was performed with very different parameter values, values chosen to mimic the behavior of cells in the cerebellum which are based on the description given in [51]–[53]. In particular, in this simulation the network topology is unchanged, but the firing behavior of the input layer was chosen to make it bursty, in imitation of the real behavior of granule cells.

**Figure 6:**
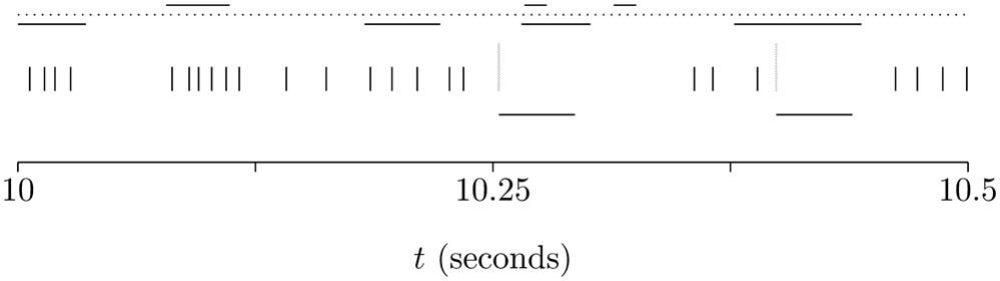
Example spike behavior. This shows the spiking activity in the Purkinje cell for one simulation trial with the parameters chosen to reflect the behavior of cells in the cerebellum. The activity between 10 s and 10.5 s is given, before plasticity has had any substantial effect. The simple spikes are marked with short black vertical dashes, the complex spikes by tall green vertical dashes; the horizontal dashes below the spikes mark the refractory periods which follow each complex spike. The horizontal lines above the spikes mark the high firing periods for the two groups, below the dotted line corresponds to the group correlated with the climbing fiber activity, above to the other group.

The firing rates of the input neurons are determined by a more complicated Cox process with two mean firing rates, a low rate 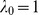 Hz and a high rate 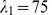 Hz. As before, the rate for each of two groups of neurons is piece-wise constant. At the start of each interval it is designated a high or low rate interval with equal probability. The actual rate at each synapse is then set as
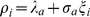
(11)where 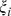 is drawn separately from a normal distribution for each input neuron in the group. If the group is beginning a high rate interval the high rate values 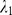 and 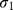 are used with probability 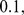 otherwise the low rate values 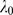 and 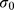 are used; for a low rate interval the low rate values are always used. The value of 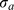 is different for low and high rate intervals chosen with 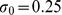 Hz and 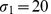 Hz. Finally, low and high rate intervals have different average length, with the low rate intervals having an average length of 75 ms and the high rate intervals an average length of 25 ms. The climbing fibre is given a firing rate using the same formula as the corresponding group but it has a higher chance of being set to the high rate: 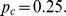 The dynamics of the synapses is the same as before. Background noise is also include as a Poisson process with rate 750 Hz, synapse strength 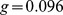 and time constant 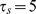 ms. An example of spiking activity in this model is given in Fig. 6.

**Figure 7:**
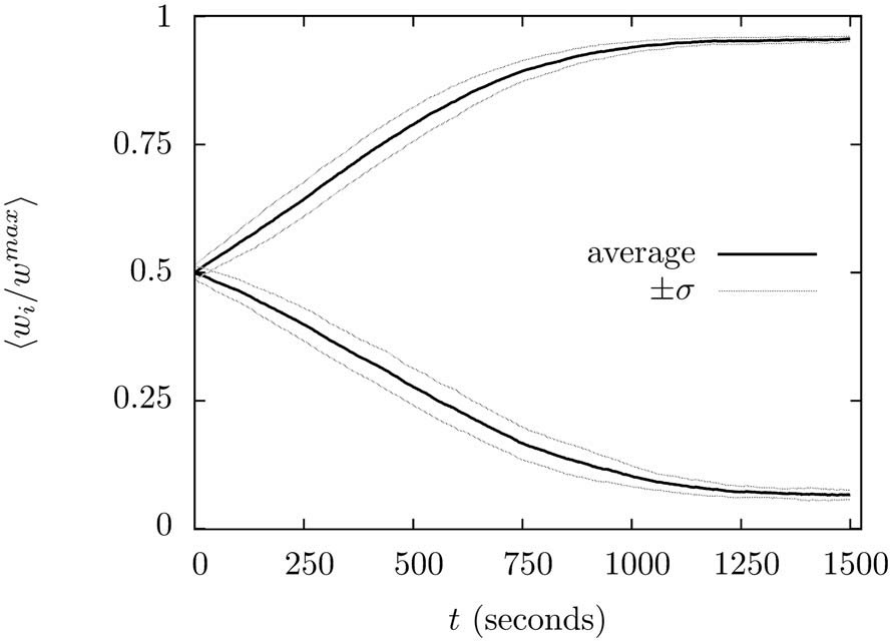
The progress of supervised learning for the granule-cell-like simulation. As before, the value of 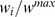 is averaged across each group during the simulation and this value is averaged over 100 trials. The black lines trace the average, the grey lines show the size of trial-to-trial variation, marking 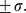

**Figure 8:**
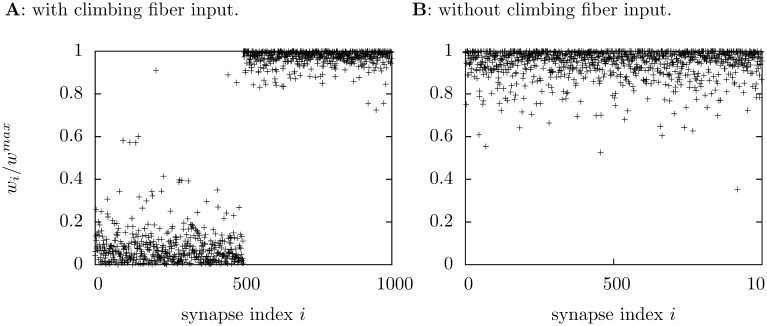
Synapse strength with and without supervision. The distribution of synapse strengths are plotted at the end of a 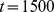 s simulation; in **A** there is climbing fiber input, in **B** there is none. In each case the first 500 synapses belong to one group and the remainder to the other.

The climbing fibre successfully supervises the learning, this can be seen in Fig. 7; the group that is correlated with the climbing fibre has an average synaptic strength 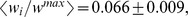 the other group has 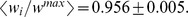 Learning occurs quickly, the low group has equilibriated after 1250 s, the other group after 1000 s. Learning does not occur without supervision; if there is no climbing fibre input the synapses in both groups equilibriate at 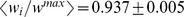 and if one group has a larger value of 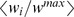 at a given time, the probability it belongs to the same group 50 s later is 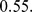 Fig. 8 shows how the distribution of synapses strengths differ in the supervized and unsupervized case.

**Figure 9:**
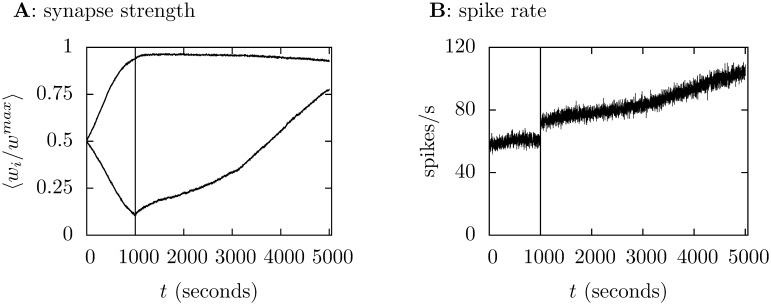
Switching off the climbing fiber. In this simulation the climbing fiber input is switched off after 1000 s, the time marked with a vertical line. **A** plots the synapse strength, the group that had been correlated with the climbing fiber input increases steadily towards the other group. The spike rate also increases, this is shown in **B**. The results are averaged over 25 trials.

The bursty input is very effective in driving the output; this is why there is no learning without the climbing fiber input: all the synapses approach the maximum value. In fact, the synapses that are suppressed by the climbing fiber input recover if that input is turned off. This is shown in Fig. 9 where the spike rate is also plotted, it increases when the climbing fiber input is switched off. This behavior is reminiscent of that observed in Purkinje cell when the inferior olive is lesioned [30], [54]–[56].

**Figure 10:**
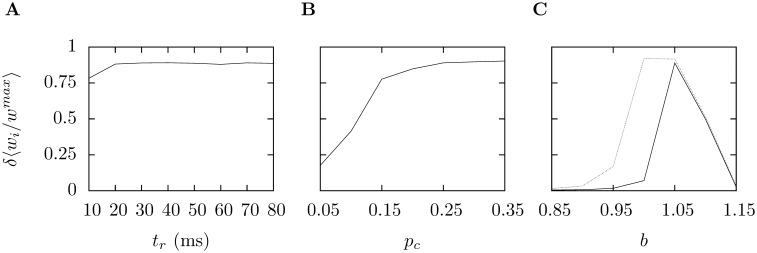
Varying the simulation parameters. In each of these graphs the difference in 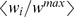 between the two groups after 1500 s is plotted. Thus, if 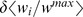 is near one the gap between the two groups is nearly as large as it could be, if it is near zero very little separates the two groups. In **A** the refractory period following a complex spike is varied between 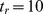 ms and 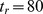 ms. For 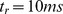 learning is reduced to 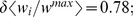 in fact the group correlated with the complex spike has 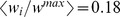 and the other group has 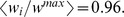 For all larger values of 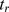 the gap 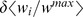 is larger than 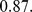 In **B**, 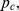 the probability that the climbing fiber is set to a high firing rate during the high firing period is varied; this does have a considerable effect on learning, at 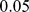 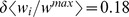 with 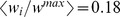 values of 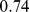 and 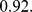 The overall firing rate of the climbing fiber changes with 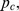 for 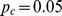 is it 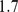 Hz, this rises to 3.3 Hz for 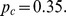 Finally, in **C** the value of 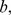 the ratio between the LTD and LTP in the STDP rule is varied. The black line plots 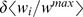 with 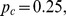 the value used elsewhere, for the grey line 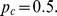 If 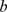 is low 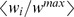 is near one for both groups, if 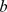 is high, it is near zero for both; learning occurs in a window between the two. In all three plots there are ten trials for each value. Supervision was successful for all but one trial for 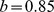 and 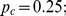 that is, in all but one trial the group correlated with the climbing fiber had a lower value of 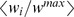 at the end of the supervision.

In these simulations the climbing fiber has a firing rate of about 2.5 Hz; this is comparable with the 1.5 Hz firing rate observed experimentally in quiescent cat [57] or the observation of a 0.9 Hz background rate in anaesthetised rat, with a high rate in response to stimulation [60]. The spiking rate of the Purkinje cell is also realistic [28], [58], [59], after learning it has an average firing rate of 62 Hz, this is the average of more and less active periods driven by the bursty input in the parallel fibers, during the more active periods it fires at 127 Hz. Changing the climbing fiber firing rate and the refractory period, 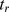, that follows complex spikes does effect learning, Fig. 10**A** and Fig. 10**B**, but learning is reasonably robust. However, learning only occurs for a small window in 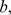 the ratio between depression and potentiation in the STDP rule, Fig. 10**C**. This window is made broader if the climbing fiber firing rate is increased, accounting for the strong depolarization associated with the complex spike by using higher values of 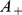 and 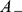 for complex spiking has a similar effect. In short, the amount of learning depends on the detail of the simulation and any precise predictions would require a much more extensive simulation. However, the increased depression observed with the higher rate of climbing fibre input is broadly consistent with the experimental observations noted in [61].

## Discussion

The example model provided here is intended to illustrate the possibility that STDP and complex spiking can result in supervized learning in a natural way. A complete account of learning in Purkinje cells will be complicated. The model presented here ignores most of the complexity of the cerebellar cortex, there are no inhibitory input cells for example. Furthermore, it ignores the complexity of the complex spike itself. The complex spike, in vivo, is generated by a broad dendritic depolarization mediated by the climbing fiber input. It is associated with calcium spikes in the dendrites which are activated by the climbing fiber input [3], [64] and these may be responsible for the sustained depolarization that causes the pause in spiking after the initial spike of a complex spike [63]. There is a very low density of sodium gates in the dendrites and they are not thought to support active back-propagation of axonal spikes, however the spikes do spread passively in the dendritic tree [5], [62], [65], [66]. The spikelets which follow the initial spike of a complex spike are, in contrast, axonally generated and, like the simple spikes, are initiated in the axonal hillock [63]. These details have been ignored here but would need to be incorporated in further development of the model. For example, the passive back-propagation of axonal spiking is likely to filter the post-synaptic spike timing. It is also likely that calcium dynamics plays a role in modulating plasticity. Finally, the treatment here ignores all but the first spike in the complex spike; it is easy to check that naïvely including the spikelets as supplementary post-synaptic spikes increases the strength of the supervisory effect since it increases the number of post-synaptic spikes in an anti-causal relationship with any pre-synaptic spikes which arrive during the subsequent refractory period.

The model uses a particularly simple description of STDP, ignoring the considerable progress that has been made in exploring different STDP rules. For example [67] presents a STDP rule which relates the plastic changes not to the post-synaptic spiking behavior but to the local post-synaptic membrane potential. This rule accounts for frequency-dependent [33], [68], [69] and dendritic-location dependent [70]–[73] effects which are observed experimentally but which are not accounted for by the STDP rule used here, despite the likely importance of dendritic-location dependent effects in the case of Purkinje cells.

It might be expected that the strong depolarization of the Purkinje cell after a complex spike would increase the size of the effect seen here. However, although STDP has been observed in Purkinje-like cells [34], little is known about what form STDP in Purkinje cells might take. Certainly, most STDP rules share an association between causal spikes and potentiation and acausal spikes and depression; the advantage of the simple rule used here is that it abstracts this common feature. What the model described here does illustrate is a potential mechanism for supervised learning with complex spikes and STDP; it remains to be seen whether this mechanism is part of learning in Purkinje cells. In fact, STDP has been previously proposed for learning in Purkinje cells: in [80] the synaptic weight distributions associated with a typical, unsupervised, STDP rule is compared with the synapse strengths for Purkinje cells and found to be in good agreement; this is certainly a criterion that could be usefully applied to more realistic STDP modelled developed from the supervised learning mechanism proposed here.

The cerebellum appears to have a role in classical conditioning [74]. This appears to support a motor learning view of cerebellar function [75] and the issue of timing, the lengthy delay between the conditioned and unconditioned stimuli, is certainly a challenge to other views of cerebellar function and for the sort of time windows normally associated with STDP. The Golgi cells are believed to act to create delays in cerebellar dynamics [76] but there is experimental evidence for a long plasticity windows directly at the parallel fiber synapses, with LTD occurring when parallel fiber input proceeds climbing fiber input by 100 ms or more [77]. The temporal ordering involved in this is the opposite to the one required for the supervized learning described here. In fact, the synaptic dynamics described in [77] are very complicated, the LTD that occurs when parallel fiber input proceeds climbing fiber input is associated with sparse parallel fiber activity; other experiments find LTD when climbing fiber activity narrowly proceeds parallel fiber activity [61], [78].

The mechanism also raises a question about the dynamics of other neurons where inhibitory input or complicated spiking dynamics which cause variable periods of quiescence: is it possible that the complex spike with its extended pause in spiking may be a particularly direct example of a more widespread phenomenon where learning is supervised through the modulation of pauses in spiking? In other words, is it possible that the simple STDP rule is a plasticity Swiss army knife, producing Hebbian and anti-Hebbian learning in different neuronal systems through the modulations of pauses? The answer seems to be no. There are brain areas that resemble the cerebellum, such as the dorsal cochlear nucleus in mammals and the electrosensory lateral line lobe of mormyrid electric fish: they have an afferent structure that is similar to the parallel fibers and they are believed to calculate a ‘negative images’, a predicted sensory background which is removed from sensory input as part of processing [79]. However, [34] indicates that the electrosensory lobe does not exploit the mechanism described here and, in fact, has a reversed STDP rule with causal spike pairs undergoing LTD. Thus, STDP appears to be variable and complicated mechanism and it remains to be seen whether the simple observation described here is useful in analysing its operation.
